# Integrative transcriptomic analysis reveals miR-26a-5p downregulation and a potential predictive gene signature for the progression of metabolic liver disease

**DOI:** 10.3389/fcell.2026.1805025

**Published:** 2026-04-20

**Authors:** Andrei Sorop, Alina-Veronica Ghionescu, Diana Larisa Ancuța, Maria-Gabriela Croitoru, Cristin Coman, Daniela Lixandru, Simona Olimpia Dima

**Affiliations:** 1 Center of Excellence in Translational Medicine, Fundeni Clinical Institute, Bucharest, Romania; 2 University of Medicine and Pharmacy “Carol Davila”, Bucharest, Romania; 3 Faculty of Biology, University of Bucharest, Bucharest, Romania

**Keywords:** DTNA, EpCAM, hepatocellular carcinoma, KPNA2, MASLD, metabolic-liver disease, microRNA, miR-26a-5p

## Abstract

**Background:**

Metabolic dysfunction–associated steatotic liver disease (MASLD) is an important inducer of hepatocellular carcinoma (HCC). MicroRNAs are key regulators of tumorigenesis. Among these, miR-26a-5p is known to be associated with liver pathogenesis, yet its role in linking MASLD progression to HCC development remains incompletely understood.

**Methods:**

Hepatic miR-26a-5p expression was quantified in the C57BL/6 mice with a 3-month high-carbohydrate diet (HCD). Public transcriptomic datasets with MASLD and HCC samples were analyzed to identify predicted miR-26a-5p downstream candidates upregulated in the above-mentioned diseases. Associations with tumor features were examined, and protein expression of β-catenin, c-MYC and EpCAM were evaluated after miR-26a-5p modulation.

**Results:**

Integrative bioinformatics identified miR-26a-5p as a candidate prognostic indicator for metabolic liver disease progression. *In vivo*, results confirmed that suppressed miR-26a-5p expression is a hallmark of diet-induced metabolic perturbation. Mechanistically, *in vitro* modulation of miR-26a-5p attenuated oncogenic signaling via the β-catenin/c-Myc/EpCAM axis, establishing its role as a tumor suppressor. Notably, *in silico* analysis of HCC tissues revealed that high miR-26a-5p levels correlate with enhanced antitumor immunity. Leveraging these insights, we constructed a transcriptional signature from miR-26a-5p downstream candidates and MASLD-HCC differentially expressed genes. This signature effectively stratifies MASLDpatients, discriminating molecular risk groups associated with progression to HCC.

**Conclusion:**

Integrating transcriptomic, clinical and experimental data suggests the role of miR-26a-5p, along with the MASLD-HCC gene signature (EpCAM, DTNA, and KPNA2), may serve as an early molecular indicator and mechanistic modulator of hepatocarcinogenesis, warranting further functional investigation.

## 1 Introduction

The global incidence of liver cancer continues to rise, representing a major public health burden (Lancet, 2025). Recently, MASLD, formerly referred to as nonalcoholic fatty liver disease (NAFLD), has emerged as a major contributor to liver pathology, with the potential to progress to HCC (Nishida, 2021; Ramirez-Mejia et al., 2024; Targher et al., 2021). Notably, MASLD-related HCC frequently arises in non-cirrhotic livers, making screening challenging and leading to delayed diagnosis and poorer survival compared with other etiologies, such as viral hepatitis (Vitellius et al., 2024; Younossi et al., 2015). Accumulating evidence indicates that excessive dietary carbohydrate intake plays a significant role in the development and progression of hepatic pathologies, alongside established risk factors such as red meat consumption and alcohol intake (Mantovani et al., 2018; Targher et al., 2021; Wahl et al., 2025). The progression from MASLD to HCC is driven by a complex interplay among innate and adaptive immune cells, hepatokines and oxidative stress, which sustain inflammation, fibrosis and a tumor-promoting hepatic microenvironment (Provera et al., 2024). Despite this growing evidence, the molecular mechanisms underlying the link between MASLD and the early stages of hepatocarcinogenesis remain to be fully elucidated. Identifying specific biomarkers that indicate the early stage of oncogenic transformation in metabolic disease may facilitate timely intervention before the clinical onset of HCC (Kutlu et al., 2018).

MicroRNAs (miRs), small RNAs of 18–22 nucleotides, are valuable tools for early detection, prognosis and monitoring of therapeutic responses due to their stability in body fluids and their ability to reflect molecular changes associated with various diseases (Angelescu et al., 2022; Ghionescu et al., 2023; Metcalf, 2024). Among these, miR-26a-5p, a miR preserved across vertebrate species (Li et al., 2019), has been reported to be involved in the development of liver pathologies (Basha et al., 2025; Cai et al., 2023; Yuan et al., 2019). We previously showed that miR-26a-5p is downregulated in type 2 diabetes and is maintained at decreased levels during progression, in MASLD and HCC, and is associated with epithelial-to-mesenchymal transition (EMT) (Ghionescu et al., 2024). Based on this evidence, we selected this molecule as a primary candidate for investigating early molecular events in metabolic liver dysfunction.

Although this molecule has been implicated in liver pathogenesis, it remains unclear whether dysregulation of this molecule contributes to the early molecular events associated with HCC. This study aimed to elucidate the impact of metabolic dysfunction associated to liver pathologies on miR-26a-5p and to use bioinformatic tools to identify its targeted gene axes relevant to disease progression. This approach may aid in the molecular stratification of patients at risk for HCC development in the metabolic context.

## 2 Materials and methods

### 2.1 Bioinformatic analysis

**FIGURE 1:**
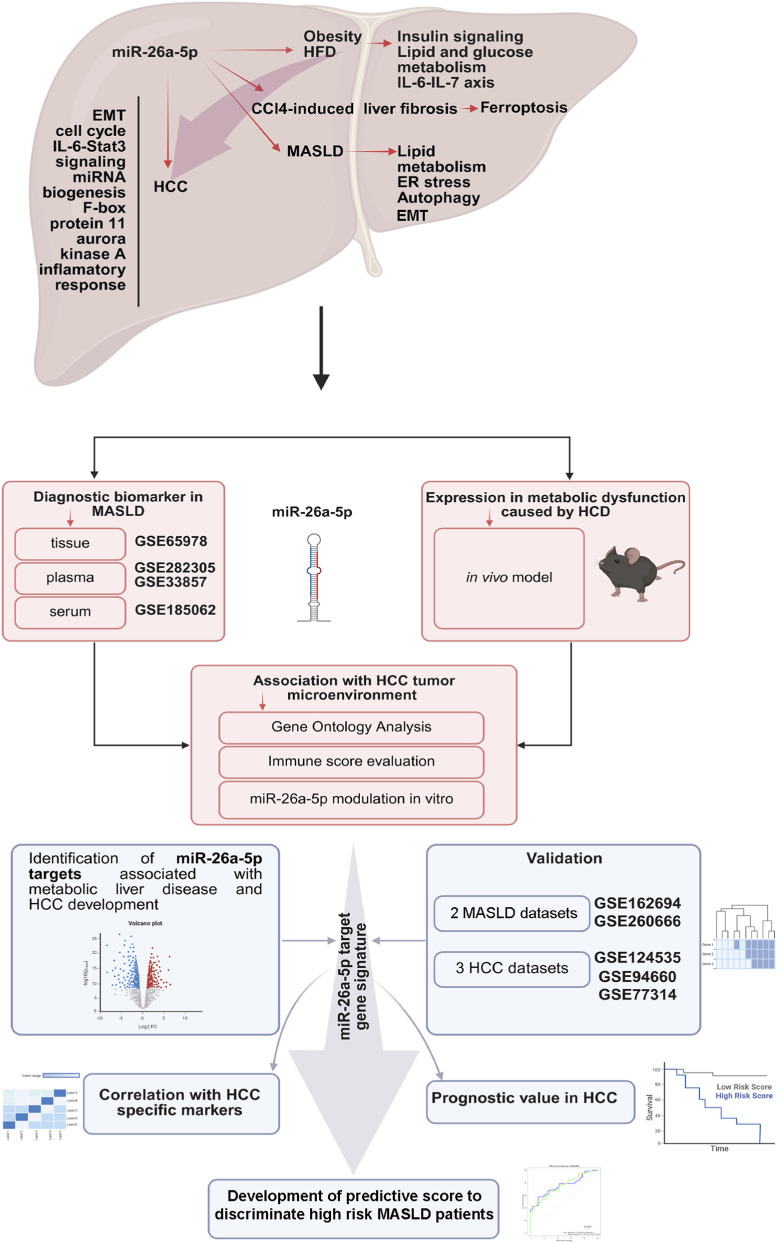
Illustration of the study workflow (created with biorender.com accessed in January 2026).

Publicly available mRNA and miR expression datasets (including clinical information) were obtained from the Gene Expression Omnibus (GEO; https://www.ncbi.nlm.nih.gov/gds) using the GEOquery R package (version 2.72), the GEO2R web-based tool (https://www.ncbi.nlm.nih.gov/geo/geo2r) (Davis and Meltzer, 2007) and The Cancer Genome Atlas (TCGA; https://portal.gdc.cancer.gov/) databases using TCGAbiolinks packages (version 2.37.2) (Colaprico et al., 2016) in the R program (version 4.4.0, https://www.R-project.org/, Vienna, Austria). The workflow of the study (integrating both experimental procedures and bioinformatic analyses based on publicly available data) is illustrated in Figure 1.

To analyse miR-26a-5p expression, we evaluated four independent publicly available datasets: GSE65978 including liver tissues from 5 ND and 5 high-fat diet (HFD) fed mice, GSE282305 (including plasma exosomes from 3 ND and 3 HFD fed mice), GSE33857 (including serum exosomes from 12 healthy volunteers and 7 MASLD) and GSE185062 (including peripheral blood samples from 10 healthy volunteers, 80 mild and 103 advanced fibrosis).

To explore miR-26a-5p downstream candidates involved in liver disease progression, we investigated for MASLD, four GEO databases: GSE126848 (14 control and 16 MASLD samples), GSE135251 (10 control and 68 MASLD), GSE260666 (6 control and 4 MASLD), GSE162694 (31 control and 47 MASLD). Moreover, for HCC, we used TCGA-Liver Hepatocellular Carcinoma (LIHC) including normal adjacent to tumor tissues (NAT; n = 50) and tumoral tissues (T; n = 371). We also included 6 GEO datasets: GSE214846 (65 NAT, 65 T), GSE207435 (27 NAT, 27 T), GSE124535 (35 NAT, 35 T), GSE94660 (21 NAT, 21 T), GSE164760 (24 NAT, 74 T) and GSE77314 (50 NAT, 50 T). We evaluated gene expression in GSE126848, as well (including liver biopsies from healthy, *n* = 14 and obese, *n* = 12).

For RNA-seq transcriptomic analyses, normalized transcripts per million (TPM) values were used for mRNA expression, while counts per million (CPM) values were used for microRNA expression. These normalized data served as the basis for downstream analyses, including comparative analyses, correlation analyses, and graphical representation, excluding differential expression analysis. Since all these datasets were collected from different sequencing platforms and methods, with unclear clinical information and various confounding variables, we analyzed each transcriptomic dataset separately to avoid false-positive results.

### 2.2 Differential expression analysis

Gene expression data (raw counts) from MASLD and HCC RNAseq datasets were used to identify differentially expressed genes (DEGs) by the DESeq2 R package (version 1.44) (Love et al., 2014). Significantly DEGs were selected based on an adjusted p-value <0.05 and an absolute log2 fold change (FC) ≥ 1.

### 2.3 *In silico* analysis to identify the predicted and validated mRNA targets

To identify miR targets, we conducted a bioinformatic analysis using the multiMIR R package (version 1.26.0, accessed in September 2025) (Ru et al., 2014) and obtained 2571 predicted and validated mRNAs. The ggvenn R package (version 0.1.19) generated the Venn diagrams, the ggplot2 R package (version 4.0.1) - the bubble plot and the pheatmap (version 1.0.13) R package–heatmaps.

### 2.4 Functional enrichment analysis of miR-26a-related DEGs in HCC

Gene Ontology (GO) - Gene Set Enrichment Analysis (GSEA) for biological processes (BP) was performed on DEGs associated with miR-26a-5p expression in TCGA-LIHC samples, using the clusterProfiler R package (version 4.12.6). GO-BP with an adjusted p-value <0.05 were considered significantly enriched.

### 2.5 *In silico* tumor microenvironment analysis

RNA-seq gene expression data were retrieved as TPM normalized counts, and miRNA expression data were obtained from the corresponding TCGA miRNA-seq datasets. Only primary tumor samples with available matched mRNA and miRNA expression profiles were included in the analysis. Gene expression values were log2-transformed prior to downstream analyses. Sample identifiers were harmonized across mRNA and miRNA datasets to ensure consistent sample matching.

The Estimation of STromal and Immune cells in MAlignant Tumors using Expression data (ESTIMATE) algorithm was applied to gene expression data in the R program to infer overall stromal and immune cell infiltration levels in TGCA-LIHC tumor samples (Sturm et al., 2020). This algorithm analyses specific gene expression signatures of immune and stromal cells (Yoshihara et al., 2013) using the immunedeconv R package (version 2.1.0) (Sturm et al., 2020).

ConsensusTME analysis was performed using the ConsensusTME R package (Jimenez-Sanchez et al., 2019) and the cancer-specific parameterization for hepatocellular carcinoma, with single-sample gene set enrichment analysis (ssGSEA) as the statistical method for computing enrichment scores.

Immune cell enrichment scores derived from ConsensusTME were visualized using heatmap-based clustering (Pearson correlation as the distance measure), enabling assessment of immune infiltration patterns across the miRNA expression gradient. Column annotations were added to display miR-26a-5p expression, immune score, stromal score, and tumor purity for each tumor sample derived from the ESTIMATE algorithm.

To characterize immune-related transcriptional programs, we performed a systematic correlation analysis between miR-26a-5p expression and a curated panel of 41 TME-associated marker genes (Lindblad and Lujambio, 2023; Seyhan et al., 2025). We included immune checkpoint molecules and cytotoxic effector genes - inhibitory immune checkpoints: hepatitis A virus cellular receptor 2 (HAVCR2 known as TIM-3), cytotoxic T-lymphocyte associated protein 4 (CTLA4), T cell immunoreceptor with Ig and ITIM domains (TIGIT), programmed cell death 1 (PD-1 known as PDCD), PD-1 ligand 1 (PD-L1 also known as CD274), PD-1 ligand 2 (PD-L2 known as PDCD1LG2), V-set immunoregulatory receptor (VSIR), indoleamine 2,3-dioxygenase 1 (IDO1), lymphocyte activating 3 (LAG3), B and T lymphocyte associated (BTLA); cytotoxic/effector T cell and natural killer (NK) cells markers: granzyme (GZM) B (GZMB), GZMA, interferon gamma (IFNG), T-box transcription factor 21 (TBX21), cluster of the differentiation (CD) 8 alpha (CD8A), CD8B, perforin 1 (PRF1), neural cell adhesion molecule 1 (NCAM1), NK cell granule protein 7 (NKG7), natural cytotoxicity triggering receptor 1 (NCR1), killer cell lectin like receptor D1 (KLRD1); regulatory T cell markers: forkhead box P3 (FOXP3), interleukin (IL) 10, IL 2 receptor subunit alpha (IL2RA), 5″-nucleotidase ecto (NT5E known as CD73), transforming growth factor beta 1 (TGFB1), ectonucleoside triphosphate diphosphohydrolase 1 (ENTPD1, known as CD39); macrophage polarization markers: CD68, mannose receptor C-type 1 (MRC1 known as CD206), CD163, arginase 1 (ARG1), nitric oxide synthase 2 (NOS2), VEGFA, prostaglandin-endoperoxide synthase 2 (PTGS2), IL6, colony stimulating factor 1 receptor (CSF1R); immunomodulatory chemokines: C-X-C motif chemokine (CXC) ligand 9 (CXCL9), CXC ligand 10 (CXCL10), CXC receptor 3 (CXCR3), C-C motif chemokine ligand (CCL) 2 (CCL2) and CCL5.

All correlations were computed using the Spearman rank analysis between hsa-miR-26a-5p expression (log_2_[CPM+1]) and the mRNA expression (log_2_[TPM+1]) of a curated panel directly from RNA-seq TCGA-LIHC tumors (*n* = 349, which are matched with the miRNA expression data available for the same tumor samples). For each gene, correlation coefficients (ρ) and corresponding p-values were calculated across all TCGA-LIHC tumor samples. To control for multiple hypothesis testing, p-values were adjusted using the Benjamini–Hochberg false discovery rate (FDR) method. Genes with FDR-adjusted p-values <0.05 were considered statistically significant.

### 2.6 Molecular risk modelling and classifier performance analysis

TPM-normalized gene expression data were obtained from the GSE193066 (106 MASLD HCC–naive patients) and GSE193080 (58 MASLD-associated HCC–experienced patients) datasets. For each cohort, samples were annotated according to molecular group (low-risk vs. high-risk for HCC) of MASLDpatients based on Prognostic Liver Signature (Fujiwara et al., 2022). All analyses were performed independently in each cohort using identical workflows.

A ridge logistic regression model was implemented using the glmnet R package. The optimal regularization parameter (λ) was determined using k-fold cross-validation implemented through the cv.glmnet function with type.measure = “auc”. Given the moderate cohort sizes, 5-fold cross-validation was applied to ensure stable estimation of model performance while maintaining sufficient observations within each fold.

To compare model performance across algorithms, a random forest classifier was implemented using the randomForest R package. The classifier performance was evaluated using receiver operating characteristic (ROC) analysis. The area under the curve (AUC) values and 95% confidence intervals were calculated using DeLong’s method. Statistical comparison of AUC between the glmnet ridge and random forest classifiers within each cohort was performed using a two-sided paired DeLong test. Calibration curves were generated using decile-based binning, and goodness-of-fit was assessed using the Hosmer–Lemeshow test.

All analyses were performed using randomForest, pROC, glmnet, ggplot2 and cowplot R packages.

### 2.7 Animals and diet

The C57BL/6 mice were obtained from the laboratory animal breeding facility of the National Institute for Medical-Military Research and Development “Cantacuzino” (CI). Then, they were acclimatized in the experimental space of the Center of Excellence in Translational Medicine, Fundeni Clinical Institute, for a minimum period of 5 days before the start of the experiment. Identification was achieved by marking the animals with markers and labelling the cages. All experimental procedures followed the provisions of international (Percie du Sert et al., 2020) and national ethics guidelines, endorsed by the ethics commission and approved by the Romanian competent authority, National Sanitary Veterinary and Food Safety Authority (number 10/20.03.2023).

The mice were divided into two groups: normal diet (ND, *n* = 4) and HCD (*n* = 4). The HCD was described previously (Ionita et al., 2022) and consisted of 69.35% nitrogen-free extract, 6.98% crude fat, 7.49% moisture, 1.42% crude ash, 0.14% crude fiber and 14.62% crude protein. The diet was administered for 90 days to establish the mouse model. Food and water were provided *ad libitum* for the entire experimental period. All aspects of animal housing and care were performed in compliance with current regulations governing animal testing. During the study, animals were housed under standard laboratory conditions in ventilated cages (Tecniplast, Buguggiate, Italy). Environmental conditions were maintained at 18–24 °C, 35%–75% relative humidity in a controlled light/dark cycle of 12 h/12 h.

### 2.8 Cell culture and transfections

Hep3B cells (European Collection of Authenticated Cell Cultures, Salisbury, United Kingdom) were grown in DMEM (1X) GlutaMAX medium, supplemented with 10% heat-inactivated fetal bovine serum, 1X non-essential amino acids solution and 50 U/mL penicillin–streptomycin (Pen-Strep, all from Gibco, Paisley, United Kingdom). For transient transfections, we used Lipofectamine 3000 (Invitrogen, Carlsbad, CA, USA) according to the manufacturer’s instructions. The cells were incubated with 50 nM hsa-miR-26a (#MC10249) or mirVana miRNA Mimic (#4464058) as a negative control for 48 h.

### 2.9 Quantitative real-time polymerase chain reaction (qRT-PCR)

Total RNA was isolated using TRIzol (Invitrogen). For mRNA, reverse transcription was performed using High-Capacity cDNA Reverse Transcription Kit (Applied Biosystems, Foster City, CA, USA) and cDNA was quantified using SYBR Green PCR Master Mix (Applied Biosystems). Ribosomal Protein S18 (Rsp18) was used as a reference gene. For miR quantification, the TaqMan MicroRNA ReverseTranscription Kit (Thermo Fisher Scientific) and TaqMan Universal PCR Master Mix, No AmpErase UNG (Thermo Fisher Scientific), were used. The primers were as follows: Rsp18 (F:5′AGGATGTGAAGGATGGGAAG3’; R:5′ACGAAGGCCCCAAAAGTG3′); vascular endothelial growth factor A (VEGFA, F:5′CTGCTGTAACGATGAAGCCCTG3’; R:5′GCTGTAGGAAGCTCATCTCTCC3′), hsa-miR-26a-5p (#000405) and U6 (#001973). All amplification reactions were performed on the 7300 Real-Time PCR System (Applied Biosystems). Relative expression levels were determined using the 2^−ΔΔCT^ method (Livak and Schmittgen, 2001; Schmittgen and Livak, 2008).

### 2.10 Western blot

Cell and tissue lysates were prepared as previously reported (Ghionescu et al., 2024). Equivalent amounts of proteins were separated via sodium dodecyl sulfate–polyacrylamide gel electrophoresis and transferred onto polyvinylidene difluoride membranes using a semi-dry transfer system (Bio-Rad, Hercules, CA, USA). Membranes were blocked with 5% non-fat dry milk (Sigma-Aldrich) and incubated with the appropriate primary antibodies following the manufacturer’s protocols. The antibodies used are as follows: rabbit anti-beta-catenin (D10A8, dilution 1:1000, #8480, Cell Signaling), mouse anti-cellular myelocytomatosis oncogene (c-Myc, 9E10, dilution 1:500, #MA1-980 Invitrogen), mouse anti-epithelial cell adhesion molecule (EpCAM, AUA1, dilution 1:500, #MA5-13917 Invitrogen), rabbit anti-glyceraldehyde 3-phosphate dehydrogenase (GAPDH, dilution 1:5000, #PA1-987 Invitrogen), goat anti-mouse (dilution 1:10000, #G-21040 Invitrogen) and goat anti-rabbit (dilution 1:10000, #G-21234 Invitrogen). Protein bands were detected by an enhanced chemiluminescence kit (ECL; Thermo Fisher Scientific-Pierce, Waltham, MA, USA) and analyzed using ImageJ software (NIH, Bethesda, MD, USA).

### 2.11 Statistical analysis

Data representation and analyses used are described in figure legends. Graphical plots were generated using the GraphPad Prism for Windows (v.10.6.1, GraphPad Software Inc., Boston, MA, United States). *p*-values < 0.05 in all tests were considered statistically significant.

## 3 Results

### 3.1 miR-26a-5p is decreased in liver pathologies

**FIGURE 2:**
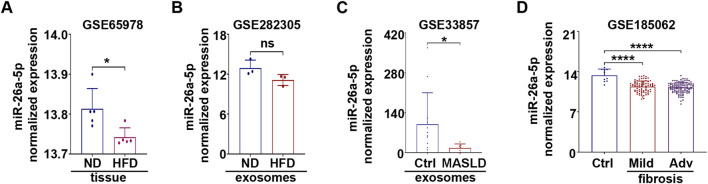
miR-26a-5p level is downregulated in metabolic-associated liver diseases. miR-26a-5p expression was evaluated in **(A)** hepatic tissue (GSE65978) and **(B)** exosomes (GSE282305) samples derived from mice fed with ND or HFD. **(C)** Exosomal miR-26a-5p expression was analyzed in MASLDpatients compared to control (Ctrl) from GSE33857. **(D)** Serum miR-26a-5p profile was evaluated in Ctrl, mild and advanced (Adv) fibrosis patients from GSE185062. Statistical significance was assessed using a nonparametric unpaired two-tailed *t*-test (*ns* - not significant, *p < 0.05, ****p < 0.0001).

First, to assess the potential of miR-26a-5p as a candidate molecular indicator, its expression levels were analyzed across different sample types using publicly available datasets. Analysis of HFD mouse liver tissues from the GSE65978 dataset revealed a significant downregulation of miR-26a-5p compared to ND controls (Figure 2A). These observations are consistent with previous reports describing reduced liver miR-26a-5p expression in HFD (Fu et al., 2015; He et al., 2017) and MASLD (Basha et al., 2025; Ghionescu et al., 2024; He et al., 2017).

Given the growing interest in circulating miRs as minimally invasive biomarkers (Roderburg and Luedde, 2014), we further examined miR-26a-5p levels in plasma-derived exosomes (GSE282305). Analysis of exosomal miRs from HFD mice showed a modest, non-significant decrease compared with controls (Figure 2B). This suggests that although hepatic miR-26a-5p is downregulated in response to HFD, its exosomal levels may be less sensitive to early metabolic alterations. Notably, exosomal miR-26a-5p expression was markedly reduced in MASLD patients’ samples from GSE33857 compared with healthy controls (Figure 2C). Given that liver fibrosis is a common consequence of metabolic disorders (Yang et al., 2025), we examined miR-26a-5p levels in serum derived from patients with fibrosis. As expected, the expression was decreased compared with that in healthy controls, independent of fibrosis stage (Figure 2D).

Collectively, these data indicate that miR-26a-5p is consistently downregulated in multiple liver pathologies, including MASLD, fibrosis and HCC, at the level of liver tissue, circulating exosomes and serum. Together, these findings support a broad association between reduced miR-26a-5p expression and liver disease progression (Ali et al., 2018; Basha et al., 2025; Cao et al., 2024; Fu et al., 2015; Fu et al., 2014; Ghionescu et al., 2024; He et al., 2017; Kota et al., 2009; Ma et al., 2018; Tian et al., 2022; Xu et al., 2021; Yang et al., 2013; Yuan et al., 2019).

### 3.2 Hepatic miR-26a-5p is reduced by a high-carbohydrate diet

**FIGURE 3:**
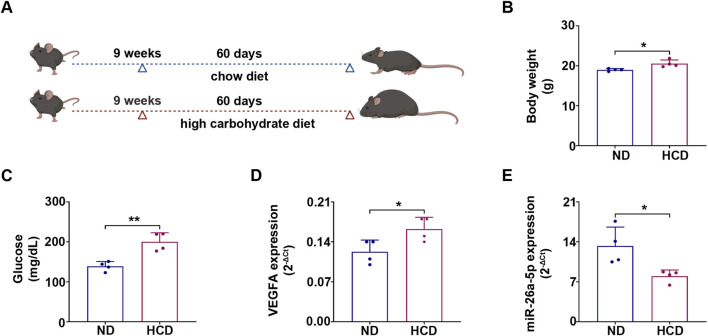
HCD alters the miR-26a-5p expression profile in mouse liver tissues. **(A)** C57BL/6J male mice were fed with HCD for 90 days. Schematic of the experimental workflow was created with biorender.com (accessed in January 2026). **(B)** Body weight and **(C)** glucose levels were measured in mice. **(D)** VEGFA mRNA levels were measured by qRT-PCR in ND and HCD liver tissues and normalized to Rsp18. **(E)** miR-26a-5p levels were detected by qRT-PCR in ND and HCD liver tissues from C57BL/6J mice and normalized to the U6 level. All barplots are presented as mean ± SD (*n* = 4). Statistical analysis was obtained by using the nonparametric unpaired t-test (*p<0.05, **p<0.01).

To determine whether carbohydrate intake, that have the same impact on developing a diet-induced metabolic perturbation as HFD, also influences hepatic miR-26a-5p expression, we next investigated HCD. Indeed, C57BL/6 mice were fed with a carbohydrate-rich diet (Figure 3A). After 90 days of dietary intervention, mice receiving HCD exhibited a modest trend towards increased body weight compared to ND controls (Figure 3B), reflecting an early stage of metabolic adaptation, along with elevated blood glucose levels (Figure 3C). In addition, hepatic expression of the angiogenic factor, VEGFA was significantly increased in HCD mice compared to the ND control group (Figure 3D), consistent with previous reports (Schuler et al., 2018). Moreover, miR-26a-5p expression was significantly decreased in HCD mice compared to ND controls as observed by qRT-PCR analysis (Figure 3E). While these findings are based on a limited sample size (*n* = 4 per group), they provide preliminary evidence that a HCD induces metabolic alterations that change hepatic miR-26a-5p expression, suggesting a potential role for this miR in early diet-associated liver pathophysiology.

### 3.3 High expression of miR-26a-5p is associated with immune-enriched TME in TCGA-LIHC

The results above prompted us to assess the clinical importance of miR-26a-5p in HCC. Therefore, based on the expression of miR-26a-5p, we stratified the tumor samples of TCGA-LIHC cohort into two different clusters based on median expression: low- (*n* = 174) and high-miR-26a-5p (*n* = 175).

To elucidate the molecular pathways underlying the role of miR-26a-5p in HCC, we performed differential gene expression analysis between the low- and high-miR-26a-5p clusters (FC > 1, *padj* < 0.01) (Supplementary Table S1), followed by functional enrichment analysis.

**FIGURE 4:**
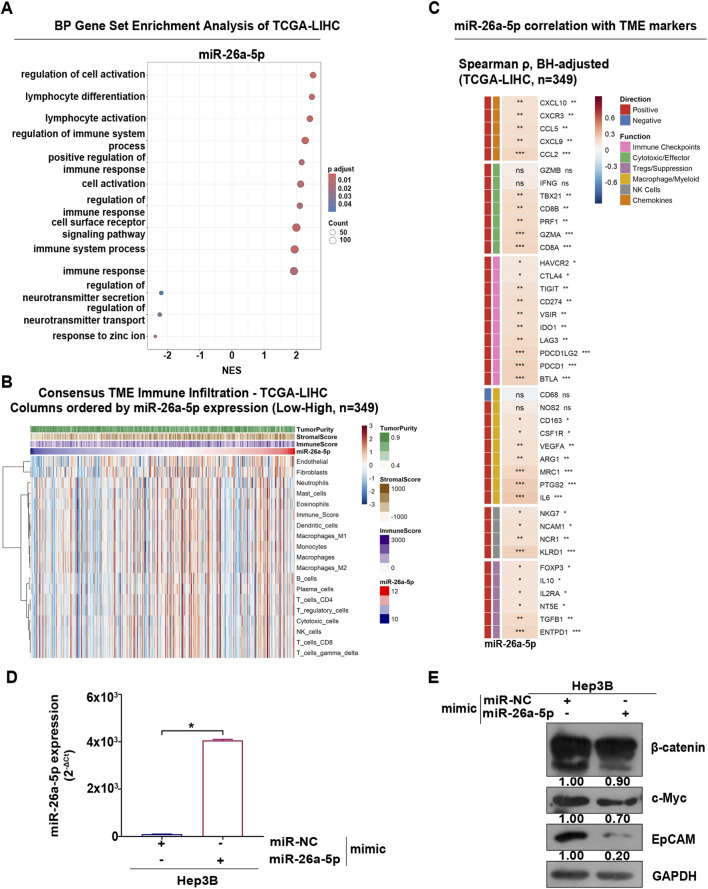
High-miR-26a-5p cluster is correlated with TME in TCGA-LIHC. **(A)** BPoverrepresentation analysis of the DEGs associated with miR-26a-5p expression in TCGA-LIHC samples identified significantly enriched BP, which are presented in a dot plot generated using the *clusterProfiler* R package. **(B)** The heatmap illustrates TME infiltration across TCGA-LIHC samples, ordered by miR-26a-5p expression. Immune and stromal cell enrichment scores were estimated from normalized gene expression data using the ConsensusTME R package (ssgsea method). Samples were arranged from low to high miR-26a-5p expression. Row-scaled enrichment scores are shown, with red indicating higher and blue lower relative infiltration. Column annotations display continuous miR-26a-5p levels and tumor microenvironment metrics (ImmuneScore, StromalScore, and TumorPurity) computed using the ESTIMATE algorithm via the immunedeconv R package. Rows were hierarchically clustered using correlation distance and Ward.D2 linkage, while column clustering was disabled to preserve the miR-26a-based sample ordering. **(C)** The heatmap shows Spearman correlations between miR-26a-5p expression and tumor microenvironment–related genes in TCGA-LIHC tumors. The gene panel included immune checkpoint genes, cytotoxic/effector T-cell markers, regulatory T-cell markers, macrophage/myeloid markers, NK-cell markers, and chemokines. Spearman correlation coefficients (ρ) were calculated for each gene, and P-values were adjusted using the Benjamini–Hochberg method. Heatmap colors indicate the strength and direction of correlation (red, positive; blue, negative). Asterisks denote adjusted significance levels (*FDR <0.05; **FDR <0.01; ***FDR <0.001). **(D)** miR-26a-5p levels in Hep3B cells transfected with either the negative control (NC) or miR-26a-5p mimic for 48 h were measured by qRT-PCR and normalized to U6. The barplots are presented as mean ± SD from two independent biological replicates. Statistical analysis was obtained by using the unpaired t-test (*p < 0.05, ***p < 0.0001, ****p < 0.0001). **(E)** Beta-catenin, c-Myc and EpCAM protein levels were measured by using the Western blot in Hep3B cells transfected with NC and miR-26a-5p mimic for 48 h. GAPDH was used as a protein loading control. Bands corresponding to the target proteins were analyzed using ImageJ and normalized to their respective internal controls. Quantification values are displayed below the panels. Data shown are representative of at least two independent experiments. Full-length blots are presented in Supplementary Material 2.

GO - GSEA for BP indicated that the high cluster was strongly associated with the upregulation of genes involved in immune response (Figure 4A; Supplementary Table S2), suggesting a potential role for miR-26a-5p in modulating the tumor microenvironment (TME), a critical factor to the tumorigenesis progression and therapeutic efficacy (Fridman et al., 2017). It can be classified according to its effect on HCC into tumor-antagonizing immune cells (such as effector T cells, CD8^+^ cytotoxic T, NK, dendritic cells, M1-polarized macrophages) and tumor-promoting immune cells (such as regulatory T cells, myeloid-derived suppressor cells, cancer-associated fibroblasts and M2-polarized macrophages) (Hao et al., 2021).

Therefore, we assessed the bulk-transcriptomic data from the TCGA-LIHC cohort, integrating miRNA expression profiles with gene expression–based estimates of immune and stromal components, to determine whether miR-26a-5p expression is associated TME of HCC. We used ESTIMATE algorithm to quantify stromal and immune cell infiltration (Yoshihara et al., 2013). Across the TCGA-LIHC cohort, miR-26a-5p expression was positively associated with both immune and stromal scores and inversely associated with inferred tumor purity. These observations suggest that tumors with higher miR-26a-5p expression are characterized by increased non-malignant cellular components within the tumor microenvironment.

To estimate enrichment scores for immune cell populations across tumor samples, we applied the ConsensusTME deconvolution framework. Visualization of immune cell signatures across tumors ordered by continuous miR-26a-5p expression revealed a progressive enrichment of multiple immune cell populations in tumors with higher miR-26a-5p levels. These immune-enriched profiles included signatures consistent with cytotoxic lymphocytes, NK cells and myeloid populations, indicating a coordinated increase in immune infiltration along the miR-26a-5p expression profile (Figure 4B).

To further explore the relationship between miR-26a-5p expression and 41 immune-related genes, we performed Spearman correlation. This analysis revealed significant positive associations between miR-26a-5p expression and multiple immune checkpoint genes (PDCD1, CD274, CTLA4 and LAG3). In addition, miR-26a-5p expression positively correlated with canonical cytotoxic T-cell markers, including CD8A, GZMB, and PRF1, as well as NK-cell–associated genes such as NKG7 and KLRD1. Furthermore, significant associations were observed with interferon-inducible chemokines involved in T-cell recruitment, including CXCL9 and CXCL10 (Figure 4C; Supplementary Table S3), which are established mediators of immune cell trafficking into tumors (Seyhan et al., 2025).

Several studies have reported that in HCC, EpCAM expression is regulated by Wingless-type (Wnt)/β-catenin signaling and c-MYC activation, thereby contributing to immune evasion (Liu et al., 2025; Yamashita et al., 2009). To investigate the effect of miR-26a-5p on these pathways, we transiently transfected Hep3B cells with miR-26a-5p mimic. Our results showed that this led to a significant decrease in the protein levels of β-catenin, EpCAM and c-MYC compared to the control (Figures 4D,E). Collectively, these results indicate that increased miR-26a-5p expression may be a modulator of the tumor microenvironment, and its downregulation in metabolic liver diseases such as MASLD, a condition reported to limit anti-tumor immune surveillance in HCC (Pfister et al., 2021), could contribute to diminished immune responses within tumors.

### 3.4 miR-26a-5p downstream candidates (EpCAM, DTNA and KPNA2) are associated with liver disease progression

To investigate the molecular mechanisms by which miR-26a-5p contributes to liver pathologies, we performed a comprehensive *in silico* analysis, integrating the overlap of DEGs in MASLD (GSE126848 and GSE135251) (Supplementary Tables S4, S5) and HCC (GSE214846 and GSE207435) (Supplementary Tables S6, S7).

**FIGURE 5:**
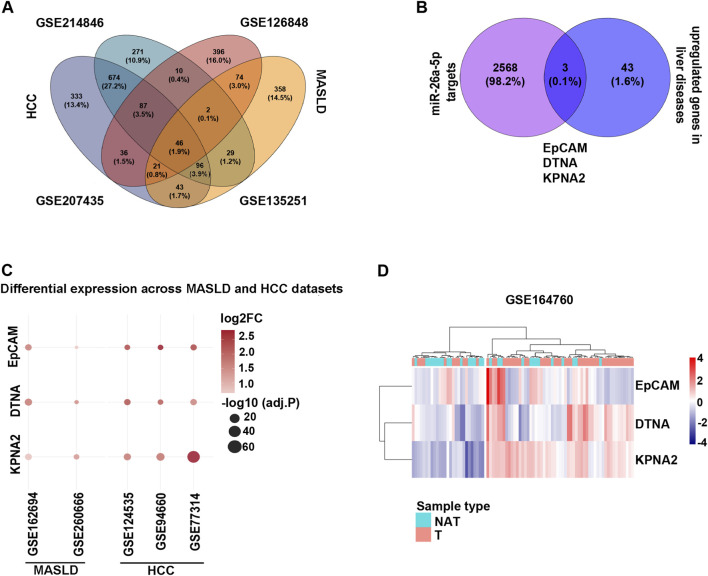
EpCAM, DTNA and KPNA2 are involved in hepatocarcinogenesis. **(A)** The Venn diagram illustrates DEGs identified from the overlap between HCC (GSE214846 and 207435) and MASLDtranscriptomic datasets (GSE126848 and GSE135251). **(B)** The Venn diagram shows genes obtained from the intersection of miR-26a-5p targets and upregulated DEGs in liver disease across the GSE214846, GSE207435, GSE126848, and GSE135251 datasets. **(C)** Bubble plot despictes the differential expression of three candidate biomarker genes—EpCAM, KPNA2, and DTNA across two independent MASLD (GSE162694 and GSE260666) and three independent HCC (GSE124535, GSE94660 and GSE77314) transcriptomic datasets. Each bubble represents a gene–dataset pair, with GEO accession identifiers on the X-axis and gene symbols on the Y-axis. Bubble size reflects statistical significance (−log10, adjusted *P* value) and color indicates log2FC. **(D)** Heatmap shows the expression profile of EpCAM, KPNA2 and DTNA in GSE164760, comparing NAT and T MASLDliver tissue. Expression values are shown as log2-transformed TPM (log2[TPM +1]) and scaled by gene (row) to highlight relative expression differences across samples. Rows correspond to genes and columns to individual samples, which are annotated by tissue status (NAT vs. T). Genes were hierarchically clustered using Euclidean distance and Ward’s linkage, while the sample order was preserved. Color intensity indicates relative expression levels, with blue denoting lower and red denoting higher expression.

We identified 46 common genes consistently upregulated across both MASLD and HCC datasets, as shown in the Venn diagram (Figure 5A; Supplementary Table S8). The presence of shared upregulated genes across disease stages indicates conserved transcriptional programs that may emerge early in chronic liver disease and persist during malignant transformation. These intersecting genes represent candidate molecular markers that link inflammatory liver pathology with hepatocarcinogenesis.

Given that miR-26a-5p is downregulated in liver disease, we focused on its predicted and validated target genes obtained using the multiMiR R package (Supplementary Table S9), which we anticipated to show increased expression. Intersecting these targets with the 46 upregulated genes yielded three overlapping candidates: EpCAM, Dystrobrevin Alpha (DTNA) and Karyopherin Subunit Alpha 2 (KPNA2) (Figure 5B).

To further validate our findings, we analyzed additional independent transcriptomic datasets, including two MASLD cohorts (GSE162694 and GSE260666) and three HCC cohorts (GSE124535, GSE94660, and GSE77314). Consistently, EpCAM, DTNA and KPNA2 were significantly upregulated across all datasets (Figure 5C; Supplementary Table S10), supporting their robustness as potential oncogenic drivers and candidate molecular indicators of hepatocarcinogenesis. Moreover, the expression of these genes remains higher in T than in NAT in MASLD -HCC patients (Figure 5D).

Notably, these genes are functionally interconnected through their involvement in EMT, stemness-associated signaling, cytoskeletal remodeling and oncogenic transcriptional programs, suggesting that miR-26a-5p loss may coordinately derepress multiple pathways driving HCC progression (Han and Wang, 2020; Hu et al., 2020; Liu et al., 2025).

### 3.5 EpCAM, KPNA2 and DTNA are correlated with HCC progression

**FIGURE 6:**
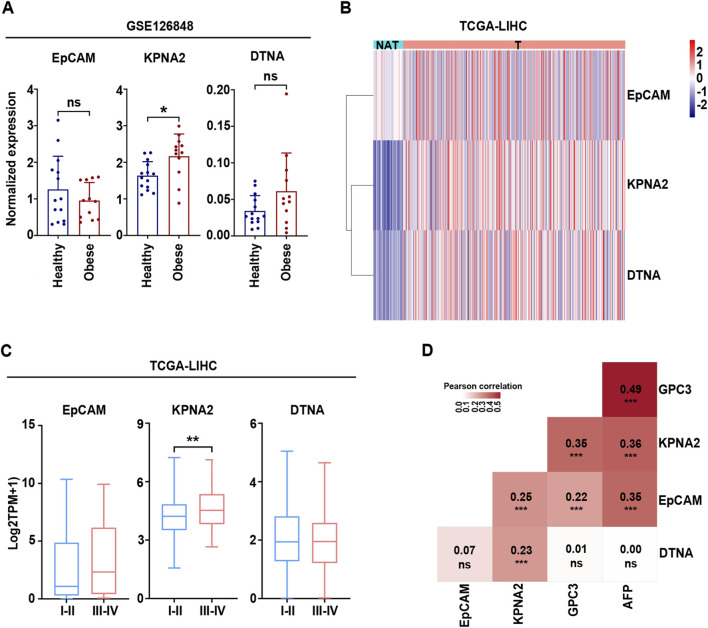
Gene-signature MASLD-HCC (EpCAM, KPNA2 and DTNA) is correlated with early metabolic disease stages and hepatocarcinogenesis. **(A)** The barplots show gene expression analysis in healthy (n = 14) and obese (n = 12) liver biopsies from GSE126848. The data are presented as mean ± SD. Statistical analysis was obtained by using the unpaired t-test. **(B)** Heatmap depicts the expression in TCGA-LIHC, comparing NAT and T liver tissue. Expression values are shown as log2-transformed TPM (log2[TPM +1]) and scaled by gene (row) to highlight relative expression differences across samples. Rows correspond to genes and columns to individual samples, which are annotated by tissue status (NAT vs. T). Genes were hierarchically clustered using Euclidean distance and Ward’s linkage, while the sample order was preserved. Color intensity indicates relative expression levels, with blue denoting lower and red denoting higher expression. **(C)** Boxplots show the expression across tumor stages in the TCGA-LIHC cohort and are presented as median values and interquartile ranges. Statistical significance was assessed using an unpaired two-tailed t-test. **(D)** The correlation matrix shows the relation between EpCAM, KPNA2, DTNA and HCC marker (GPC3 and AFP). The Pearson correlation was used for statistical analysis (ns = not significant, *p < 0.05, **p < 0.01, ***p < 0.0001, ****p < 0.0001).

Our analysis of publicly available RNA data indicated that EpCAM, KPNA2 and DTNA were upregulated in HCC samples. Given the strong association between MASLD and obesity (Quek et al., 2023), we assessed gene expression at early disease stages in overweight individuals and found that hepatic KPNA2 expression was elevated in obese patients compared with normal-weight controls (Figure 6A). To further investigate their potential associations with clinicopathological features, we explored the TCGA-LIHC cohort. These results confirmed the coordinated upregulation of EpCAM, KPNA2, and DTNA in T compared to NAT and validated this three-gene signature as a robust transcriptional marker of hepatic tumors (Figure 6B). Moreover, to explore the role of these genes in HCC progression, we evaluated their expression at different cancer stages, defining the extent of disease and reflecting whether the tumor has spread locally or to distant sites (Duseja, 2014). Patients were stratified into early-stage (stages I–II) and late-stage (stages III–IV) groups. Notably, KPNA2 expression progressively increased with advancing tumor stage, suggesting a potential role in hepatocarcinogenesis (Figure 6C). Moreover, EpCAM, KPNA2 and DTNA showed positively correlations with one another, and both KPNA2 and EpCAM with HCC markers, including alpha-fetoprotein (AFP) and glypican 3 (GPC3), as well (Figure 6D).

### 3.6 EpCAM, KPNA2 and DTNA could classify the molecular risk groups associated with liver disease progression

**FIGURE 7:**
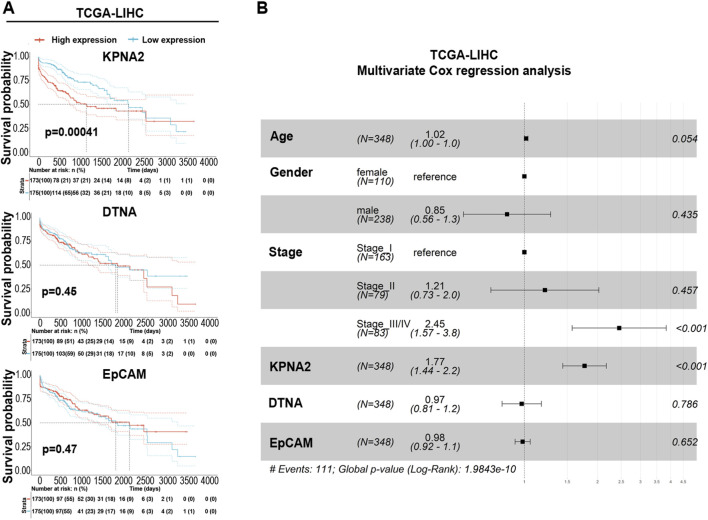
KPNA2 is correlated with a poor prognosis in HCC patients. **(A)** Kaplan-Meier survival curves for the TCGA-LIHC cohort, stratified into high- and low-groups based on median KPNA2, DTNA, and EpCAM expression levels. The cutoff point was the median risk score. 95% confidence intervals are shown by shaded ribbons. A statistically significant difference in overall survival between high- and low-expression groups is indicated by the log-rank test p-value. The corresponding risk table shows the number and proportion of patients at risk over time. **(B)** Forest plot indicating multivariate Cox proportional hazard analysis to evaluate the hazard ratio of our selected genes KPNA2, DTNA and EPCAM in MASLD-HCC for overall survival among clinicopathological features in the TCGA-LIHC cohort. After adjusting for all other features, each horizontal line indicates the predictor’s hazard ratio (HR) and 95% confidence interval (CI). The survival analysis was performed by using the survival 3.5.8 and survminer 0.4.9 R packages.

To further investigate the functional impact of the gene signature, TCGA-LIHC patients were stratified into high- and low-expression groups based on the median expression of each gene. Kaplan–Meier survival analysis revealed that only high KPNA2 expression was significantly associated with poorer overall survival (Figure 7A). These findings were further confirmed by multivariate Cox regression analysis, supporting its potential role as a prognostic marker (Figure 7B).

**FIGURE 8:**
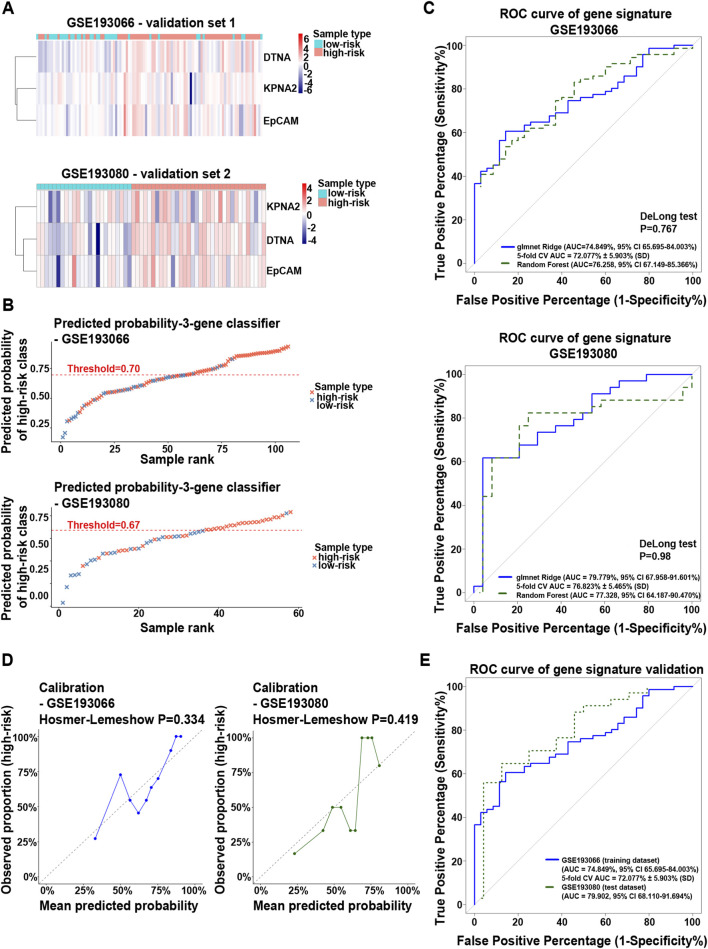
Performance of gene signature (EpCAM, KPNA2 and DTNA) in MASLD-associated HCC in two independent public transcriptomic datasets. **(A)** Heatmaps depict the profile expression in GSE193066 and GSE193080, comparing high- and low-risk HCC patients. The heatmap was generated from log2-transformed TPM (log2[TPM +1]) expression values and scaled by row (z-score normalization) to highlight relative expression differences across samples. Rows represent individual genes and columns represent samples, annotated by sample type. Genes were hierarchically clustered using Euclidean distance and Ward’s linkage, while the sample order was preserved. Color intensity indicates relative expression levels, with blue denoting lower and red denoting higher expression. **(B)** The plots illustrate the ranked predicted probability for the glmnet Ridge classifier in GSE193066 (MASLD HCC-naïve patients) and GSE193080 (MASLD HCC–experienced patients). Each symbol represents one sample, coloured by assigned molecular risk class (red - high-risk; blue-low-risk). The red dashed line indicates the Youden-index optimal threshold, derived independently for each cohort. **(C)** ROC curves show the 3-gene molecular risk classifier for GSE193066 and GSE193080 (blue: glmnet Ridge, green dashed: random Forest). Both classifiers were fitted independently for each cohort. AUC values with 95% confidence intervals (DeLong method) and 5-fold CV AUC ±SD are shown and a statistical comparison between ROC curves was performed using a two-sided DeLong test. **(D)** The figure indicates the calibration curves for the glmnet Ridge classifier in GSE193066 (blue) and GSE193080 (green). Each point represents one decile of predicted probabilities (X-axis: mean predicted probability; Y-axis: observed proportion of high-risk samples). Grey dashed diagonal: perfect calibration. Hosmer–Lemeshow P-values indicate adequate calibration in both cohorts. **(E)** The combined ROC plot illustrates the performance of the glmnet Ridge classifier across three analytical stages. A three-gene classifier was developed using a Ridge-penalized logistic regression model (glmnet, α = 0), with 5-fold cross-validation conducted on the GSE193066 training dataset (shown in blue). The fitted model, with coefficients set at the cross-validated optimum (λ_min), was subsequently applied to the test GSE193080 dataset (shown in green) without any retraining or recalibration. The ROC analysis demonstrates strong discriminatory performance in the external cohort, indicating that the model maintains predictive accuracy across different datasets. In the plot, the solid blue line represents the ROC for the discovery cohort, while the dotted dark green line represents the ROC for the external test cohort.

Building on the prognostic significance of KPNA2, we explored the predictive value of this three-gene signature for HCC development in MASLD patients. We used independent cohorts, including both MASLD-HCC-naïve (GSE193066) and MASLD-HCC-experienced (GSE193080) patients. Initially, the expression profiles of EpCAM, KPNA2 and DTNA were evaluated, showing higher expression in high-risk patients than in low-risk individuals in both transcriptomic datasets (Figure 8A).

To evaluate the discriminative capacity of the three-gene signature (KPNA2, DTNA and EpCAM), we developed and validated a penalized logistic regression classifier using Ridge regularization with the glmnet R package. This analysis was conducted across two independent transcriptomic cohorts: GSE193066 and GSE193080 (Supplementary Figure S1). The analysis followed a sequential three-stage framework: (i) standalone performance assessment within each cohort, including internal cross-validation and calibration; (ii) head-to-head comparison with a Random Forest classifier; and (iii) external validation in which the model trained exclusively on GSE193066 was applied without modification to GSE193080.

As an initial characterisation of classifier output, predicted probabilities of high-risk class membership were computed for all samples in each cohort and ranked from lowest to highest. In both GSE193066 and GSE193080, the probability ranking plots reveal a separation between molecularly defined low-risk and high-risk samples across the probability spectrum, with the large majority of low-risk samples accumulating below the optimal decision threshold (Youden-index) (Figure 8B).

To quantify the discriminative ability of the ridge regression and random forest classifiers, ROC analysis was performed independently for each cohort (Figure 8C). In the GSE193066, the glmnet Ridge classifier achieved an AUC of 74.8% (95% CI 65.7%–84.0%), with a 5-fold cross-validated AUC of 72.1% ± 5.9% (SD). The Random Forest classifier showed a comparable AUC of 76.3% (95% CI 67.1%–85.4%). In GSE193080, where both classifiers were refitted and the glmnet Ridge model achieved an AUC of 79.8% (95% CI 68.0%–91.6%) with a 5-fold cross-validated AUC of 76.8% ± 5.5% (SD) and the random forest classifier yielded an AUC of 77.3% (95% CI 64.2%–90.5%). The replication of the discriminatory signal in an independently fitted model on a separate dataset strengthens confidence in the biological validity of this three-gene signature. Calibration analysis confirmed that the predicted probabilities from the glmnet Ridge model were discriminative and closely aligned with observed high-risk proportions (Hosmer–Lemeshow test, = 0.334 and *p* = 0.419, respectively) (Figure 8D).

To confirm the results, we applied formal external validation, in which the glmnet ridge model-including the regularisation parameter, all three gene coefficients and the Youden-index threshold of 0.696-was locked from the discovery cohort and applied without any modification to GSE193080. The external validation achieved an AUC of 79.9% (95% CI 68.1%–91.7%), the highest across all three analytical stages (Figure 8E).

The convergence of consistent AUC estimates indicates that KPNA2, DTNA and EPCAM collectively constitute a potential molecular classifier across independent transcriptomic datasets. Importantly, all three genes are absent from the Fujiwara/Hoshida signature gene panel (Supplementary Figure S2; Supplementary Table S11), indicating that the classifier captures a biologically independent signal rather than recapitulating the label-derivation framework.

## 4 Discussion

HCC is an increasingly recognized major complication of MASLD. Although lifestyle interventions remain the primary preventive approach (Lange et al., 2021), current strategies are insufficient, highlighting the urgent need to define the molecular and tumorigenic mechanisms that drive disease progression. Extensive studies have shown that hepatic miR-26a plays a central role in regulating glucose, lipid metabolism and insulin signaling. The upregulation of this molecule effectively prevents obesity-induced metabolic disturbances, promoting miR-26a as a potential therapeutic target for metabolic syndrome (Ali et al., 2018; Fu et al., 2015). Moreover, Jin F. et al. showed that miR-26 induces apoptosis by inhibiting autophagy, thereby increasing chemosensitivity in HCC cells (Jin et al., 2017). Previously, we showed that miR-26a-5p is downregulated in early stages of type 2 diabetes-associated metabolic dysfunction and remains suppressed during progression. Also, it broadly regulates multiple EMT-related genes, including N-cadherin, linking diabetes-related liver impairment to HCC development (Ghionescu et al., 2024).

In the present study, we observed that an HCD and HFD markedly reduced hepatic miR-26a-5p expression in mice, suggesting a potential role for this miR in mediating diet-associated liver pathophysiology. Reduced expression was also noticed in MASLD and HCC tissue samples. Moreover, reduced miR-26a-5p was found in circulating exosomes from patients with MASLD or fibrosis. These results suggest that loss of miR-26a-5p is a recurrent event during liver disease progression and it is tempting to hypothesize that this molecule is a candidate molecular indicator for MASLD-associated injury and HCC.

A previous study demonstrated that miR-26a inhibited IL-17 and IL-6 *in vivo* and *in vitro*. IL-6 overexpression completely reversed miR-26a′s protective effects on liver weight, triglyceride accumulation and serum alanine aminotransferase levels, highlighting the miR-26a–IL-6–IL-17 axis as a key regulator of MASLD development in mice (He et al., 2017). These findings prompted us to investigate the immune-regulatory role of miR-26a-5p in HCC.

Based on immune deconvolution using the ConsensusTME analysis in the TCGA-LIHC cohort, we observed that tumors with high miR-26a-5p expression exhibited increased immune-stromal infiltration and lower tumor purity. These observations are particularly relevant in the context of metabolic-associated HCC, in which impaired anti-tumor immune surveillance has been reported (Pfister et al., 2021).

In addition, miR-26a-5p expression was positively correlated with multiple immune regulatory and effector genes (CD8A, PDCD1, PDCD1LG2, KLRD1) and the T-cell-recruitment chemokines (CXCL9 and CXCL10), which are canonical features of interferon-driven immune-inflamed tumor microenvironments. Interferon-γ signaling and its downstream chemokines CXCL9 and CXCL10 play a central role in the recruitment of cytotoxic T lymphocytes into tumors and have been implicated in shaping immune-active HCC phenotypes that may respond to immune checkpoint blockade (Chen et al., 2023; Ruiz de Galarreta et al., 2019). However, because bulk transcriptomic analyses capture composite signals from mixed cellular populations, the observed association between miR-26a-5p expression and immune-related transcripts may reflect differences in tumor cellular composition rather than tumor-intrinsic regulation of immune pathways. Accordingly, our results should be interpreted as indicating that miR-26a-5p abundance tracks with immune-enriched HCC microenvironments, rather than demonstrating a direct immunomodulatory role. Future studies using single-cell transcriptomics or spatial profiling approaches will be necessary to determine the cell-type-specific origin of miR-26a-5p expression and to clarify its potential role in shaping the immune architecture of HCC tumors.

Moreover, miR-26a-5p overexpression suppressed β-catenin, c-MYC and EpCAM expression in HCC cells. Activation of the Wnt/β-catenin pathway is a well-established driver of immune evasion in HCC, promoting tumor cell stemness and limiting immune cell infiltration (Liu et al., 2025; Yamashita et al., 2009).

To further explore the downstream effectors, we performed an integrative *in silico* analysis and identified three downstream candidates of miR-26a-5p (EpCAM, DTNA and KPNA2) that were consistently upregulated across MASLDand HCC. These molecules are involved in key biological processes that promote liver disease progression. EpCAM is an important marker for HCC (Liu et al., 2025) and in advanced cirrhosis, the EpCAM-expressing cells with stemness properties, mediated by Wnt signaling, have been identified as key factors for hepatocarcinogenesis (Khosla et al., 2017). Moreover, DTNA has been shown to promote signal transducer and activator of transcription 3/transforming growth factor beta signaling while it inhibits P53 activity (Hu et al., 2020). KPNA2, in turn, is an inducer of telomerase activity (Ding et al., 2025) and has also been reported to affect P53 expression (Tang et al., 2022). In addition, KPNA2 has previously been included in diagnostic prediction panels with significance in MASLDand HCC, together with the mediator complex subunit 8 and Y-Box Binding Protein 1, further supporting the relevance of this gene set in liver disease progression (Ni et al., 2024). Many studies have identified different miRs that sponge KPNA2, including miR-411-5p (Chen et al., 2021) and miR-26b (Lin et al., 2018; Tang et al., 2022). Dysregulation of these pathways promotes hepatocarcinogenesis and metabolic dysfunction, supporting the concept that miR-26a-5p acts as a multifaceted tumor suppressor and metabolic regulator in the liver.

Our results show that KPNA2 increases in the early stage of disease, in obese patients and remains upregulated in advanced stages. Importantly, MASLDpatients which high risk for HCC also have heightened levels. We also observed that this gene was associated with HCC stage and markers, such as AFP and GPC3, and was an independent prognostic factor for patients’ survival. Moreover, our findings indicate that the MASLD-HCC signature (EpCAM, DTNA and KPNA2) classifies molecular risk groups, supporting its potential as a candidate prognostic indicator in this clinical setting.

Although our study provides an important foundation for subsequent wet-lab validation studies in HCC biology, several limitations should be considered. While the relatively small sample size in the *in vivo* HCD model was sufficient to identify statistically significant changes in hepatic miR-26a-5p expression that align with our *in silico* findings, it is underpowered to capture the full spectrum of transcriptomic and histological changes. Consequently, future studies with larger cohorts and longitudinal histological assessment are warranted to fully characterize the role of the miR-26a-5p-related axes across the progression from diet-induced metabolic perturbation to HCC. Even if *in vitro* and *in vivo* data support a functional role for miR-26a-5p, further validation using genetic loss- and gain-of-function models is required.

Moreover, although an integrative *in silico* analysis identified three downstream candidates of miR-26a-5p (EpCAM, DTNA, and KPNA2) that were consistently upregulated in MASLD and HCC and the protein expression analysis for EpCAM showed a trend consistent with our transcriptomic data, future studies will validate these axes biochemically, which will be essential to fully elucidate the regulatory network of miR-26a-5p in the context of diet-induced liver stress. In addition, while the MASLD-HCC signature defined by these genes appears to reliably stratify patients by HCC risk, its predictive value requires confirmation in prospective clinical studies.

In summary, our findings suggest that miR-26a-5p and its downstream candidates (EpCAM, DTNA and KPNA2) may reflect molecular transitions between metabolic dysregulation and HCC. These associations provide a basis for further investigation into their potential utility for risk stratification and targeted intervention strategies in progression of MASLD.

## Data Availability

The original contributions presented in the study are included in the article/Supplementary Material, further inquiries can be directed to the corresponding authors.

## Glossary

Adv: Advanced
AFP: Alpha-fetoprotein
AUC: Area under the curve
ARG1: Arginase 1
BP: Biological processes
BTLA: B and T lymphocyte associated
c-Myc: Cellular myelocytomatosis oncogene
CCL: C-C motif chemokine ligand
CD: Cluster of the differentiation
CSF1R: Colony stimulating factor 1 receptor
CTLA4: Cytotoxic T-lymphocyte associated protein 4
Ctrl: Control
CXC: C-X-C motif chemokine
DEGs: Differentially expressed genes
DTNA: Dystrobrevin Alpha
EMT: Epithelial-to-mesenchymal transition
EpCAM: Epithelial cell adhesion molecule
ER: Endoplasmic reticulum
ESTIMATE: Estimation of STromal and Immune cells in MAlignant Tumours using Expression data
ENTPD1: Ectonucleoside triphosphate diphosphohydrolase 1
FC: Fold change
FDR: False discovery rate
FOXP3: Forkhead box P3
GAPDH: Glyceraldehyde 3-phosphate dehydrogenase
GO: Gene Ontology
GSEA: Gene set enrichment analysis
GPC3: Glypican 3
GZM: Granzyme
HCC: Hepatocellular carcinoma
HCD: High-carbohydrate diet
HFD: High-fat diet
HAVCR2: Hepatitis A virus cellular receptor 2
IDO1: Indoleamine 2,3-dioxygenase 1
IFNG: Interferon gamma
IL: Interleukin
IL2RA: IL 2 receptor subunit alpha
KLRD1: Killer cell lectin like receptor D1
KPNA2: Karyopherin Subunit Alpha 2
LAG3: Lymphocyte activating 3
LIHC: Liver Hepatocellular carcinoma
MASLD: Metabolic dysfunction–associated steatotic liver disease
miRs: MicroRNAs
MRC1: Mannose receptor C-type 1
NAFLD: Nonalcoholic fatty liver disease
NAT: Normal adjacent to tumor tissue
NCAM1: Neural cell adhesion molecule 1
NCR1: Natural cytotoxicity triggering receptor 1
ND: Normal diet
NK: Natural killer
NKG7: NK cell granule protein 7
NOS2: Nitric oxide synthase 2
NT5E: 5″-nucleotidase ecto
PD-1: Programmed cell death 1
PD-L1: Programmed cell death 1 ligand 1
PD-L2: PD-1 ligand 2
PRF1: Perforin 1
PTGS2: Prostaglandin-endoperoxide synthase 2
ROC: Receiver operating characteristic
Rsp18: Ribosomal Protein S18
TCGA: The Cancer Genome Atlas
T: Tumor
TBX21: T-box transcription factor 21
TGFB1: Transforming growth factor beta 1
TIGIT: T cell immunoreceptor with Ig and ITIM domains
TME: Tumor microenvironment
TPM: Transcripts per million
VEGFA: Vascular endothelial growth factor A
VSIR: V-set immunoregulatory receptor
Wnt: Wingless-type
