# Supplementary figures and images for: Integrative transcriptomic analysis reveals miR-26a-5p downregulation and a potential predictive gene signature for the progression of metabolic liver disease
Source: Front Cell Dev Biol. 2026 Apr 20;14:1805025. doi: 10.3389/fcell.2026.1805025 (PMC13136239; doi:10.3389/fcell.2026.1805025)

The full uncropped images of the Western blot (Figure 4E)

$\beta$ -catenin

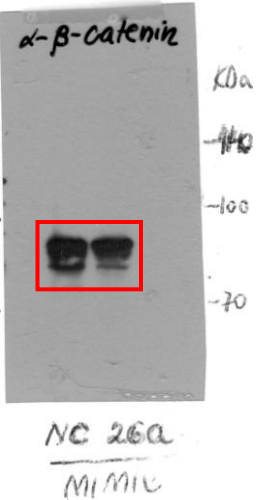

EpCAM

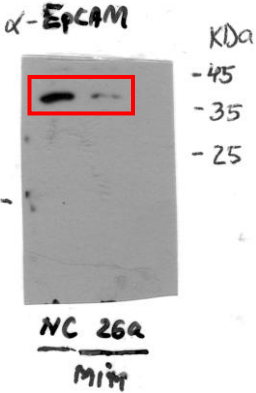

c-Myc

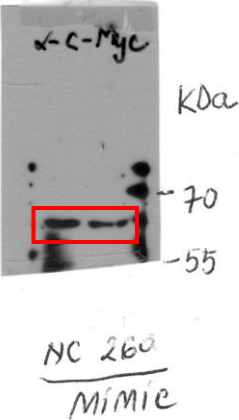

GAPDH

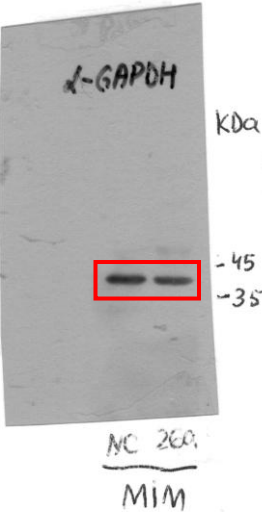

Supplement: Supplementary file 1 [file DataSheet2.pdf]
