# Supplementary material for: Integrative transcriptomic analysis reveals miR-26a-5p downregulation and a potential predictive gene signature for the progression of metabolic liver disease
Source: Front Cell Dev Biol. 2026 Apr 20;14:1805025. doi: 10.3389/fcell.2026.1805025 (PMC13136239; doi:10.3389/fcell.2026.1805025)
Supplement: Supplementary file 2 [file DataSheet1.pdf]

Supplementary material

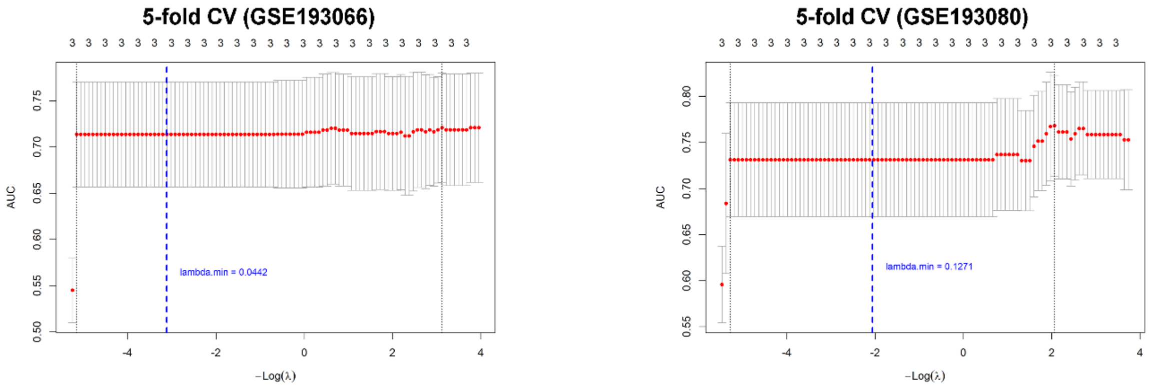

Supplementary Figure 1. The image illustrates the cross-validation analysis used to select the optimal regularization parameter in ridge-penalized logistic regression models for GSE193066 and GSE193080. 5-fold cross-validation curves showing the relationship between model performance and the regularization parameter (λ) for the three-gene classifier. The mean cross-validated area under the receiver operating characteristic curve (AUC) is plotted against −log(λ), with red points representing the average AUC across folds and grey bars indicating the standard error. The dashed blue vertical line denotes the optimal regularization parameter (λ_min) corresponding to the maximum cross-validated performance. The selected λ values were used to derive the final ridge-regularized logistic regression models for downstream risk classification analyses.

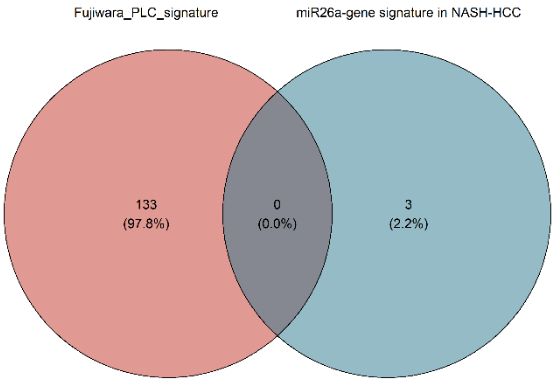

Supplementary Figure 2. **Comparison between the Fujiwara prognostic liver signature and the miR-26a–associated gene signature.** The Venn diagram shows the overlap between genes included in the previously described Fujiwara et al prognostic liver signature (PLS) and the miR-26a–associated gene signature identified in MASLD-HCC liver tissue. Gene symbols from both signatures were intersected and visualized using the ggvenn package in R. Each circle represents the total number of genes within the respective signature, and the overlapping region indicates genes shared between the two gene sets. Percentages correspond to the proportion of genes shared relative to the total number of genes in each signature. The limited overlap between the two signatures indicates that the miR-26a–associated gene panel represents a molecular program largely distinct from the Fujiwara PLS framework.
